# A Novel Non-Invasive Approach Based on Serum Ceruloplasmin for Identifying Non-Alcoholic Steatohepatitis Patients in the Non-Diabetic Population

**DOI:** 10.3389/fmed.2022.900794

**Published:** 2022-06-20

**Authors:** Qingling Wang, Da Zhou, Mingjie Wang, Mingyu Zhu, Peizhan Chen, Hu Li, Meng Lu, Xinxin Zhang, Xizhong Shen, Taotao Liu, Li Chen

**Affiliations:** ^1^Department of Gastroenterology, Ruijin Hospital, Shanghai Jiao Tong University, School of Medicine, Shanghai, China; ^2^Medical School, Kunming University of Science and Technology, Kunming, China; ^3^Department of Gastroenterology and Hepatology, Zhongshan Hospital of Fudan University, Shanghai, China; ^4^Shanghai Institute of Liver Diseases, Fudan University Shanghai Medical College, Shanghai, China; ^5^Central Laboratory, Ruijin Hospital, Shanghai Jiao Tong University, School of Medicine, Shanghai, China; ^6^Department of Infectious Disease, Shanghai Jiao Tong University Affiliated Sixth People‘s Hospital, Shanghai, China; ^7^Research Laboratory of Clinical Virology, Department of Infectious Disease, Ruijin Hospital, Shanghai Jiao Tong University, School of Medicine, Shanghai, China

**Keywords:** non-alcoholic fatty liver disease, non-alcoholic steatohepatitis, ceruloplasmin, non-invasive diagnosis, logistic models

## Abstract

**Background and Aim:**

Few non-invasive models were established to identify patients with non-alcoholic steatohepatitis (NASH). Liver biopsy remains the gold standard in the clinic. Decreased serum ceruloplasmin (CP) is reported in patients with non-alcoholic fatty liver disease (NAFLD). We aimed to develop a non-invasive model incorporating CP for identifying NASH from NAFLD without type 2 diabetes mellitus (T2DM).

**Methods:**

A total of 138 biopsy-proven patients with NAFLD without T2DM were enrolled. The CP ratio was calculated for standardization as the CP value divided by the lower limit of normal. The clinical, anthropometric, biochemical, and histological parameters were compared between the low and high CP ratio groups divided by the median value. Multivariate logistic regression analysis was performed to develop a model for identifying NASH in patients with NAFLD.

**Results:**

The medians of the high (*n* = 69) and low (*n* = 69) CP ratio groups were 1.43 (1.28–1.61) and 1.03 (0.94–1.12), respectively. A comparison of the two groups showed that the severity of steatosis, hepatocellular ballooning, inflammation activity, fibrosis, and liver iron deposition decreased along with the CP ratio (*p* < 0.05). The median CP ratio of patients with NASH was significantly lower than those with NAFL [1.15 (1.01–1.41) *vs*. 1.33 (1.24–1.54), *p* = 0.001]. A novel model which consists of the CP ratio, BMI, and aspartate aminotransferase (AST) was developed. The AUCs of the model in discriminating NASH from NAFLD was 0.796 (0.694–0.899) and 0.849 (0.713–0.984) in the training and validation groups, and 0.836 (0.659–1.000), 0.833 (0.705–0.962), and 0.821 (0.612–1.000) in patients with normal serum alanine aminotransferase, AST, and both levels, respectively.

**Conclusions:**

Decreased CP ratio is associated with more severe histological activity, a diagnosis of NASH, and hepatic iron deposition among patients with NAFLD without T2DM. The CP ratio model could be served as a non-invasive approach to identifying patients with NASH, which might reduce the need for liver biopsy.

## Introduction

Non-alcoholic fatty liver disease (NAFLD) is emerging as the most common cause of end-stage liver disease that affects about 30% of the general adult population globally (1). Compared to benign non-alcoholic fatty liver disease (NAFL), non-alcoholic steatohepatitis (NASH) is the necro-inflammatory entity of NAFLD and accounts for 20–27% of patients having NAFLD (2). Liver fibrosis develops more rapidly in patients with NASH, which is likely driven by necroinflammation (3). The incidence of cirrhosis and hepatocellular carcinoma in patients with NASH is much higher compared to that in patients with NAFL (4). Moreover, NASH is more commonly associated with multiple extrahepatic complications, including cardiovascular disease and extrahepatic malignancy (5) as well as an increased risk of mortality (6). Therefore, it is very important to identify patients with NASH who represent the risk population urgent to intervention in the clinic. To date, live biopsy remains the gold standard to diagnose NASH, whereas the invasive nature of the procedure leading to bleeding, infection, and even death limits its application (7). Increased aminotransferase has a low diagnostic value because some patients with biopsy-proven NASH have normal levels (4). The cytokeratin 18 fragment is the most validated parameter, while its diagnostic performance is still unsatisfactory (8). Existing clinical models (9–12) are unable to be widely applied ascribed to the derivation from morbidly obese patients or high complexity in the calculation. Thus, a simpler, more convenient, and non-invasive approach is urgently needed to identify NASH individuals from NAFLD.

Serum ceruloplasmin (CP) is mainly synthesized and secreted by hepatocytes (13). The CP is an important multicopper-containing protein and transports copper through the body. The copper metabolic disorders can induce CP deficiency, such as Wilson's disease due to impaired copper mobilization (14). CP also serves as a ferroxidase that oxidizes ferrous iron to ferric iron for further incorporation into transferrin (15). Despite the important diagnostic value in Wilson's disease, some studies suggested serum CP was inversely correlated to fibrosis or positively associated with steatosis in hepatitis B patients (16, 17). And another study enrolled 100 children with biopsy-proven NAFLD demonstrated that CP had an accuracy of 82% in distinguishing pediatric patients with NAS score ≥ 5 from those with NAS score < 5 (18). However, the clinical significance and association of CP with the severity of NAFLD are unclear in the adult population. It is necessary to evaluate the potential application of CP as a diagnostic biomarker in the clinic.

In terms of metabolic dysfunction, NAFLD is deemed as an earlier event before type 2 diabetes mellitus (T2DM) and has great value in predicting T2DM even independent of all other components of Metabolic syndrome (MetS) (19). On the contrary, T2DM promotes the progression of NAFLD and elevates the risk of cirrhosis (20, 21). The histopathology of NAFLD patients with T2DM is always more severe and recognizable to be intervened earlier in the clinic. Thus, identifying NASH at a relatively earlier stage of NAFLD is of more clinical significance. Therefore, we evaluated the demographic, metabolic, and histological data retrospectively in a Chinese group of liver biopsy-proven NAFLD patients without T2DM. The aims of the present study were to explore the association of serum CP with the histological severity of NAFLD and to examine whether CP alone or combined with other variables can be used to predict the presence of NASH.

## Patients And Methods

### Study Population

A total of 297 patients with liver biopsy-proven NAFLD from November 2018 to February 2022, were enrolled retrospectively. Among them, 218 patients came from Ruijin Hospital, Shanghai Jiaotong University and 79 patients came from Zhongshan Hospital, Fudan University. The inclusion criteria were as follows: (1) patients whose ages were no <18 years old; (2) those who had complete values of statistical variables; (3) patients without T2DM. The exclusion criteria were patients with excessive alcoholic uptake (>30 g/day for men or 20 g/day for women) (5) or those coexisting with polycystic ovary syndrome and other liver diseases, such as Wilson's disease, hepatitis B/C virus infection, and drug-induced liver injury (22). This study was approved by the Institutional Ethics Committee of Shanghai Ruijin Hospital and Zhongshan hospital (B2020-085) and complied with the Helsinki Declaration of 1964 and later versions. All patients signed the informed consent before liver biopsy.

### Data Collection and Laboratory Assessment

The demographic characteristics and medical history were obtained from patient interviews during screening. The information on ophthalmological examination using a slit lamp for each patient was recorded on the day of liver biopsy. The laboratory tests of all blood samples were performed in each clinical laboratory. The serum CP was detected with commercial reagents by an automatic biochemistry analyzer with a lower limit of normal as 200 mg/ml in the Shanghai Ruijin Hospital (AU5800, Beckman Coulter, USA), or as 150 mg/ml in the Zhongshan Hospital of Fudan University (7,600, HITACHI, Japan). Thus, to balance the intra-laboratory difference in CP detection, the CP ratio was calculated for standardization as the CP value divided by the lower limit of normal of each laboratory.

T2DM was diagnosed when subjects with fasting blood glucose ≥ 7.0 mmol/L, or glycated hemoglobin A1c ≥ 6.5% (23). Hypertension was determined when patients presented with blood pressure ≥ 140/90 mmHg twice measured on different days. MetS was confirmed (24) when patients with at least three of the following points: (1) waistline ≥ 85 and 80 cm for men and women; (2) hypertension, or systolic blood pressure ≥ 130 mmHg or diastolic blood pressure ≥ 85 mmHg; (3) T2DM, or fasting blood glucose ≥ 5.6 mmol/L; (4) triglyceride ≥ 1.7 mmol/L, or specific drug treatment; (5) high-density lipoprotein <1.0 and 1.3 mmol/L for men and women, or specific drug treatment.

### Histopathology Evaluation

All liver samples were analyzed blindly by two experienced pathologists with the Steatosis-Activity-Fibrosis (SAF) scoring algorithm (25). At the same time, the score of hepatocellular ballooning was evaluated directly. According to the SAF scoring algorithm, NAFLD was diagnosed when the hepatocellular steatosis ≥5% without other etiologies histologically (26), while NASH was defined as at least 1 point of each following histological feature: hepatic steatosis, hepatocellular ballooning, and lobular inflammation (25). The presence of hepatic copper or iron deposition in each liver sample was analyzed by Rhodanine or Perl's dyeing. For those patients whose diagnostic scores (27) were more than two points, ATP7b gene sequencing was further performed to exclude Wilson's disease.

### Statistical Analysis

The qualitative variables were shown as frequencies and percentages and analyzed using Pearson's Chi-square test. The continuous variables were described as medians and interquartile ranges and analyzed using the Student's *t*-test and analysis of variance, or Mann–Whitney *U* and Kruskal–Wallis *H* tests when appropriate. Multivariable logistic regression analysis was performed to create a model for identifying NASH from NAFLD. The diagnostic performance was analyzed by the receiver operator characteristic curve (ROC) analysis and compared using the Delong test. The analyses and the figures were performed using the IBM SPSS software (version 22.0, USA), GraphPad Prism (version 8.4.3, USA), or R software (version 4.1.2, USA). Two-sided *p* < 0.05 was considered to be statistically significant. In all figures, *p* < 0.05 was shown as ^*^, *p* < 0.01 was ^**^, *p* < 0.001 was ^***^, and null was not significant.

## Results

### General Characteristics and Biochemical Parameters of the Study Population

**Figure 1:**
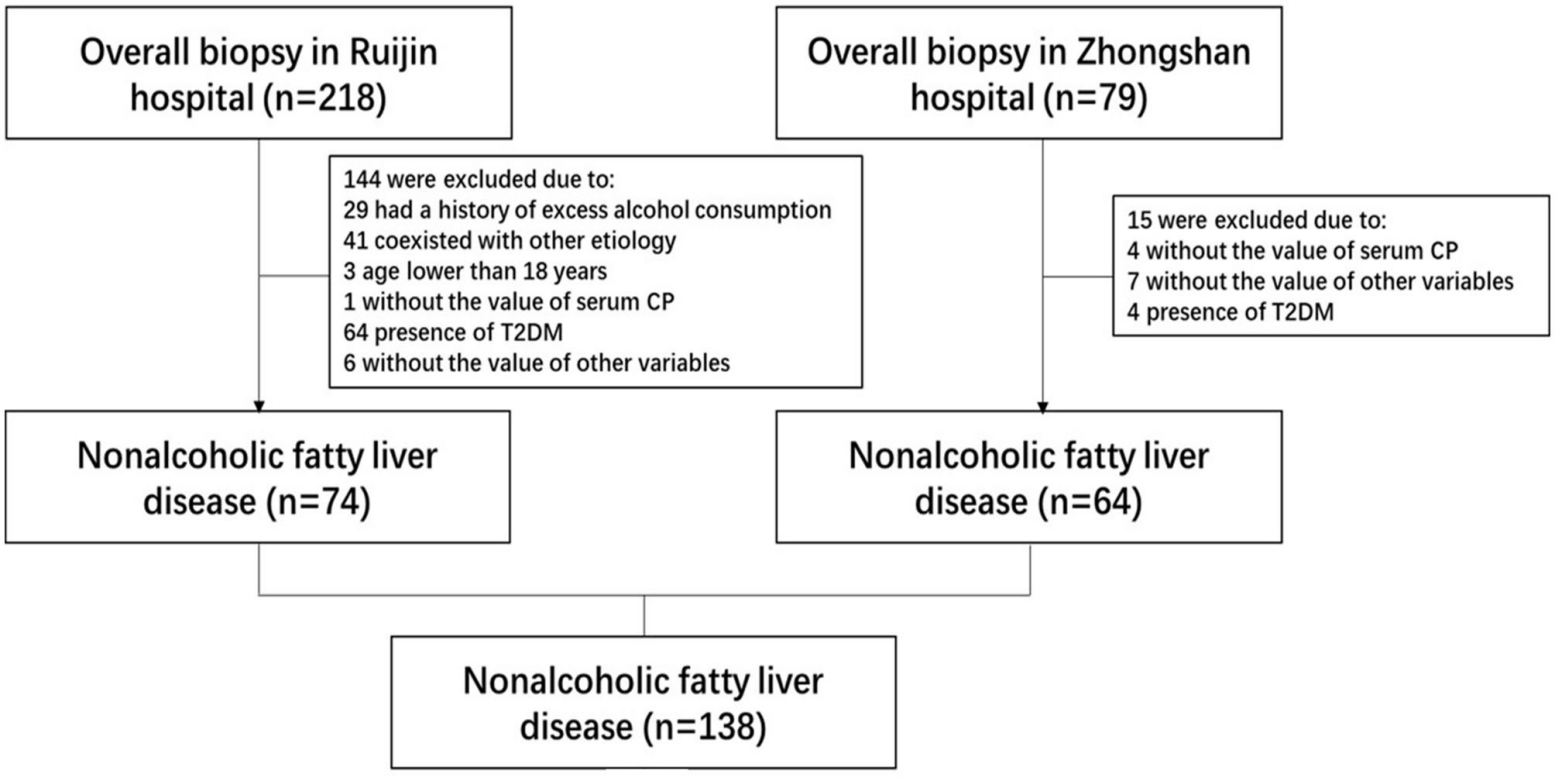
The flow chart for patient inclusion.

A total of 138 adult patients with NAFLD were eventually enrolled in the present study. The flow chart of patients' enrollment is shown in Figure 1. The general characteristics of patients were summarized in Table 1. Among the overall group, the median age and body mass index (BMI) of patients were 39.00 (33.00–54.25) years and 27.41 (24.67–30.87) Kg/m^2^, while 84 (60.9%) patients were male. Hypertension was present in 46 (33.3%) patients and MetS was in 65 (47.1%) patients. The medians serum CP and CP ratio were 211.00 (186.00–236.63) mg/L and 1.19 (1.03–1.43), while 28 (20.3%) patients showed decreased CP level [177.85 (159.23–192.35) mg/L], and 110 (79.7%) exhibited normal CP level [215.65 (200.60–241.15) mg/L].

**Table 1:** The characteristics of patients in the low and high CP ratio groups.

**Variables**	**Overall (*n* = 138)**	**CP ratio ≥1.19 (*n* = 69, 50%)**	**CP ratio <1.19 (*n* = 69, 50%)**	***p*** **-Value**
Hypertension, *n* (%)	46 (33.3)	25 (36.2)	21 (30.4)	0.470
MetS, *n* (%)	65 (47.1)	37 (53.6)	28 (40.6)	0.125
Male, *n* (%)	84 (60.9)	35 (50.7)	49 (71.0)	0.015
age, (years)	39.00 (33.00–54.25)	39.00 (33.50–54.00)	42.00 (33.00–54.50)	0.998
BMI, (Kg/m2)	27.41 (24.67–30.87)	27.62 (24.59–31.3)	27.36 (24.65–30.55)	0.435
CP, (mg/L)	211.00 (186.00–236.63)	232.00 (208.50–260.00)	197.20 (172.50–213.90)	0.000
CP ratio	1.19 (1.03–1.43)	1.43 (1.28–1.61)	1.03 (0.94–1.12)	0.000
ALT, (IU/L)	88.00 (50.75–128.25)	93.00 (56.50–128.50)	84.00 (44.00–128.50)	0.457
normal ALT, *n* (%)	34 (24.6)	13 (18.8)	21 (30.4)	0.114
AST, (IU/L)	48.00 (32.00–66.00)	48.00 (32.50–64.50)	48.00 (30.00–68.00)	0.681
normal AST, *n* (%)	61 (44.2)	26 (37.7)	35 (50.7)	0.123
normal ALTAST, *n* (%)	31 (22.5)	12 (17.4)	19 (27.5)	0.153
AKP, (IU/L)	74.00 (62.75–89.25)	75.00 (62.00–91.00)	73.00 (63.50–89.00)	0.997
GGT, (IU/L)	60.00 (35.00–87.00)	67.00 (45.00–88.00)	55.00 (33.50–85.50)	0.182
UA, (μmol/L)	412.00 (324.75–487.08)	416.00 (333.00–479.35)	408.00 (312.85–488.80)	0.946
Creatine, (umol/L)	76.50 (65.00–89.50)	71.50 (59.25–89.50)	77.50 (70.25–89.25)	0.198
TG, (mmol/L)	1.70 (1.22–2.20)	1.68 (1.18–2.17)	1.79 (1.26–2.35)	0.436
CHOL, (mmol/L)	4.92 (4.19–5.59)	4.97 (4.16–5.74)	4.91 (4.18–5.45)	0.388
HDL, (mmol/L)	1.06 (0.96–1.22)	1.04 (0.93–1.19)	1.07 (0.98–1.23)	0.317
LDL, (mmol/L)	3.00 (2.60–3.68)	2.94 (2.56–3.71)	3.14 (2.65–3.67)	0.797

*AKP, alkaline phosphatase; ALT, alanine transferase; AST, aspartate transferase; BMI, body mass index; CHOL, cholesterol; CP, ceruloplasmin; GGT, gamma-glutamyl transferase; HDL, high density lipoprotein; LDL, low density lipoprotein; MetS, metabolic syndrome; TG, triglyceride; UA, uric acid*.

In order to explore the difference between patients with different CP ratio levels, patients were divided into low and high CP ratio groups equally [69 (50.0%) *vs*. 69 (50.0%)] according to the median of the CP ratio of 1.19. The medians of the high and low CP ratio groups were 1.43 (1.28–1.61) and 1.03 (0.94–1.12), respectively. The proportion of males in the low CP ratio group was significantly greater than that in the high CP ratio group (71.0% *vs*. 50.7%, *p* = 0.015). The differences in other clinical, anthropometric, and biochemical parameters were not significant between the two groups (*p* > 0.05, Table 1).

### The Association of the CP Ratio With the Histological Severity of NAFLD

**Figure 2:**
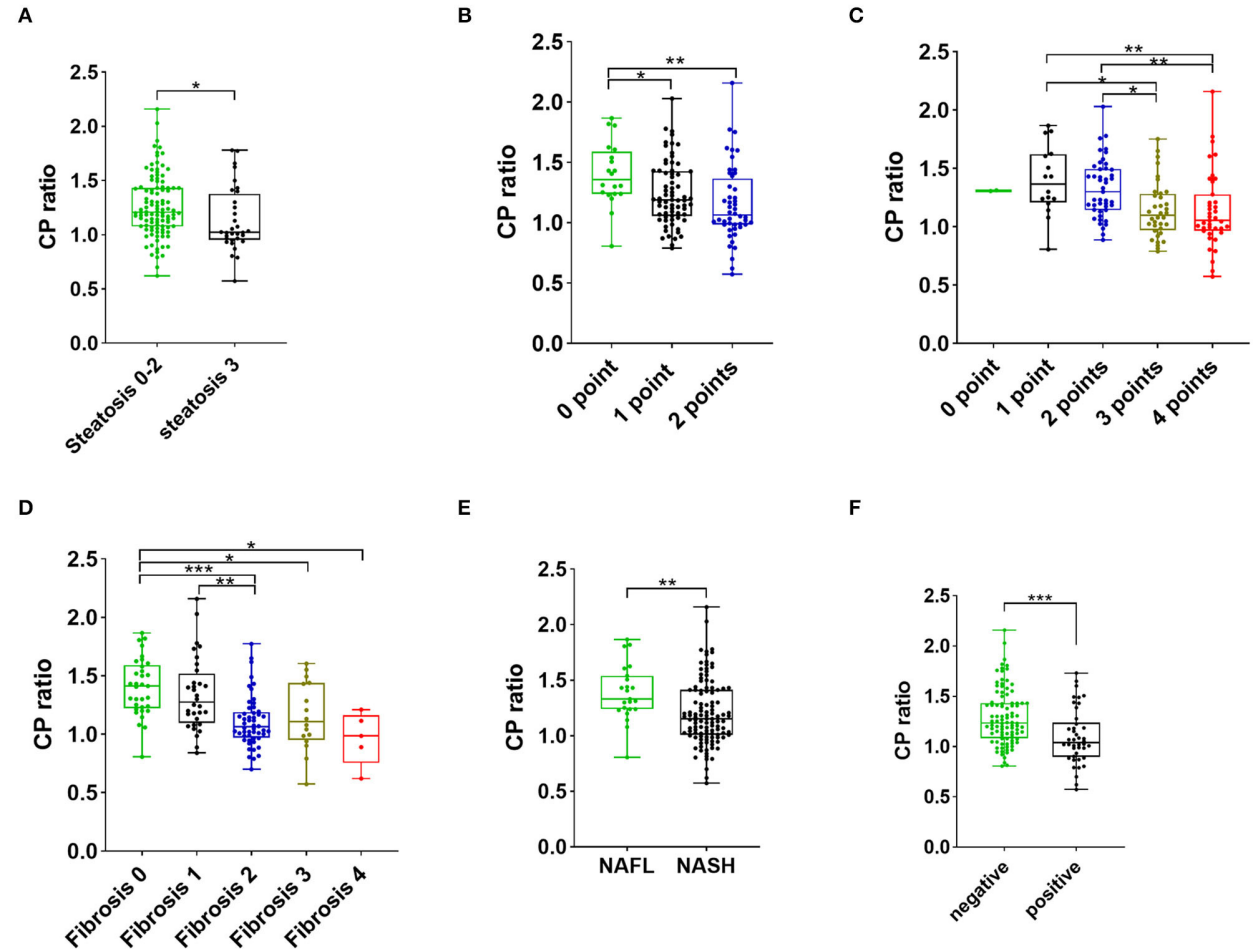
The CP ratio of patients with steatosis stages 0–2 vs. 3 **(A)**, with different scores of ballooning **(B)**, with various scores of inflammation activity **(C)**, with different grades of fibrosis **(D)**, with NAFL and NASH **(E)**, and with negative and positive Perl's staining **(F)**. The value within the box ranges from the 25^th^ to the 75^th^ percentiles. The median is shown by the horizontal bar. The vertical bars represent the values between the minimum and the maximum. The * indicates as *p* < 0.05, ** as *p* < 0.01, and *** as *p* < 0.001. Abbreviations: CP, ceruloplasmin; NAFL, non-alcoholic fatty liver; NASH, non-alcoholic steatohepatitis.

The distribution of patients at various histological stages of NAFLD is listed in Table 2. The proportion of steatosis >66% in the low CP ratio group was significantly greater than that in the high CP ratio group (30.4% *vs*. 15.9%, *p* = 0.044). The median CP ratio of patients with steatosis >66% was significantly lower than that of patients with steatosis ≤ 66% [1.02 (0.95–1.38) *vs*. 1.21 (1.08–1.43),*p* = 0.01, Figure 2A].

**Table 2:** The histopathological features of patients in the low and high CP ratio groups.

**Variables**	**Overall (*n* = 138)**	**CP ratio ≥1.19 (*n* = 69, 50%)**	**CP ratio <1.19 (*n* = 69, 50%)**	***p*** **-Value**
Steatosis, *n* (%)				–
0	4 (2.9)	4 (5.8)	0 (0.0)	
1	51 (37.0)	28 (40.6)	23 (33.3)	
2	51 (37.0)	26 (37.7)	25 (36.2)	
3	32 (23.2)	11 (15.9)	21 (30.4)	
Steatosis 1–3, *n* (%)	134 (97.1)	65 (94.2)	69 (100.0)	0.128
Steatosis 2–3, *n* (%)	83 (60.1)	37 (53.6)	46 (66.7)	0.118
Steatosis 3, *n* (%)	32 (23.2)	11 (15.9)	21 (30.4)	0.044
Ballooning, *n* (%)				0.000
0	20 (14.5)	18 (26.1)	2 (2.9)	
1	70 (50.7)	35 (50.7)	35 (50.7)	
2	48 (34.8)	16 (23.2)	32 (46.4)	
Ballooning 1–2, *n* (%)	118 (85.5)	51 (73.9)	67 (97.1)	0.000
Ballooning 2, *n* (%)	48 (34.8)	16 (23.2)	32 (46.4)	0.004
Activity, *n* (%)				0.000
0	2 (1.5)	2 (2.9)	0 (0.0)	
1	16 (11.6)	13 (18.8)	3 (4.4)	
2	45 (32.6)	30 (43.5)	15 (21.7)	
3	36 (26.1)	12 (17.4)	24 (34.8)	
4	39 (28.3)	12 (17.4)	27 (39.1)	
Activity 1–4, *n* (%)	136 (98.6)	67 (97.1)	69 (100.0)	0.154
Activity 2–4, *n* (%)	120 (87.0)	54 (78.3)	66 (95.7)	0.002
Activity 3–4, *n* (%)	75 (54.4)	24 (34.8)	51 (73.9)	0.000
Activity 4, *n* (%)	39 (28.3)	12 (17.4)	27 (39.1)	0.005
Fibrosis, *n* (%)				0.000
0	33 (23.9)	28 (40.6)	5 (7.2)	
1	32 (23.2)	20 (29)	12 (17.4)	
2	52 (37.7)	13 (18.8)	39 (56.5)	
3	16 (11.6)	7 (10.1)	9 (13.0)	
4	5 (3.6)	1 (1.4)	4 (5.8)	
Fibrosis 1–4, *n* (%)	105 (76.1)	41 (59.4)	64 (92.8)	0.000
Fibrosis 2–4, *n* (%)	73 (52.9)	21 (30.4)	52 (75.4)	0.000
Fibrosis 3–4, *n* (%)	21 (15.2)	8 (11.6)	13 (18.8)	0.236
Fibrosis 4, *n* (%)	5 (3.6)	1 (1.4)	4 (5.8)	0.362
NAFL, *n* (%)	23 (16.7)	20 (29.0)	3 (4.3)	0.000
NASH, *n* (%)	115 (83.3)	49 (71.0)	66 (95.7)	0.000
Positive Perl's staining, *n* (%)	42 (30.4)	11 (15.9)	31 (44.9)	0.000

*CP, ceruloplasmin; NASH, nonalcoholic steatohepatitis*.

The hepatocellular ballooning of patients with low CP ratio was more severe than that of patients with high CP ratio. The proportions of hepatocellular ballooning 1–2 points (97.1% *vs*. 73.9%, *p* < 0.001) and 2 points (46.4% *vs*. 23.2%, *p* = 0.004) in the low CP ratio group were significantly greater than those in the high CP ratio group. The CP ratio was inversely associated with the severity of ballooning (Figure 2B).

The proportions of inflammation activity 2–4 points (95.7% *vs*. 78.3%, *p* = 0.002), 3–4 points (73.9% *vs*. 34.8%, *p* < 0.001) and 4 points (39.1% *vs*. 17.4%, *p* = 0.005) were significantly greater in the low CP ratio group than those of the high CP ratio group. From 1 to 4 points of inflammation activity, the CP ratio significantly decreased (Figure 2C).

The proportions of the presence of fibrosis (fibrosis stage 1–4, 92.8% vs. 59.4%, *p* < 0.001) and significant fibrosis (fibrosis stage 2–4, 75.4% vs. 30.4%, *p* < 0.001) in the low CP ratio group were higher than those in the high CP ratio group. Furthermore, the CP ratio was inversely correlated to the severity of fibrosis (Figure 2D).

Based on the diagnostic criteria of histopathological NASH, 23 (16.7%) patients were diagnosed as NAFL, while 115 (83.3%) patients were diagnosed as NASH. Compared to patients with a high CP ratio, the proportion of NASH was higher in patients with a low CP ratio (95.7% *vs*. 71.0%, *p* < 0.001). The median CP ratio of patients with NASH was significantly lower than that of patients with NAFL [1.15 (1.01–1.41) *vs*. 1.33 (1.24–1.54), *p* = 0.001, Figure 2E].

### The Correlation of the CP Ratio With Hepatic Iron Deposition

The proportion of liver iron overload, indicated by positive hepatic Perl's staining, in NAFLD patients with low CP ratio was significantly greater than that in those with high CP ratio (44.9% *vs*. 15.9%, *p* < 0.001). The median CP ratio of patients with positive Perl's staining was significantly lower than that of patients with negative staining [1.04 (0.90–1.24) *vs*. 1.24 (1.08–1.43), *p* < 0.001, Figure 2F].

### The Development of the CP Ratio Model in Discriminating NASH

**Figure 3:**
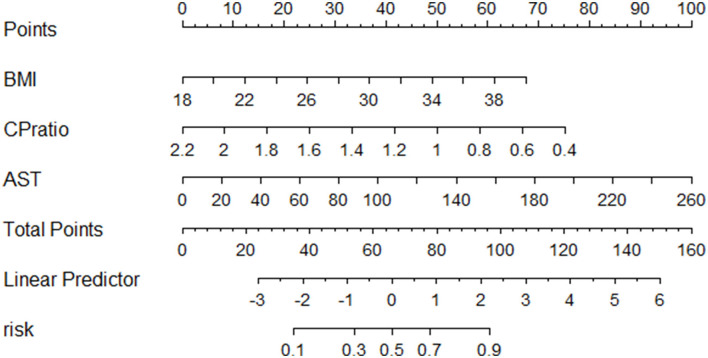
The nomogram of the CP model for discriminating NASH. Abbreviations: AST, aspartate transferase; BMI, body mass index; CP, ceruloplasmin.

The patients were 70% sampled randomly as the training group by using the R package randomizeR (28) to develop a novel predictive model for NASH, while the rest 30% patients as the validation group. The differences in statistical variables between the two groups were not statistically significant (Supplementary Tables 1, 2). By using univariate analysis in the training group, the male sex, CP ratio, BMI, alanine aminotransferase (ALT), aspartate aminotransferase (AST), uric acid, and triglyceride were associated with NASH (*p* < 0.05, Table 3). Then the multivariable logistic regression analysis found that the CP ratio [odds ratio (OR): 0.051, 95% confidence interval (CI): 0.006–0.432, *p* = 0.006], BMI (OR: 1.245, 95%CI: 1.062–1.460, *p* = 0.007), and AST (OR: 1.028, 95%CI: 1.002–1.054, *p* = 0.034) were independently associated with the presence of NASH (Table 3). There was no linear relationship among the three continuous variables. The novel model was devised as follows: CP ratio model =1/ {1 + exp [– (−2.086 + 0.219^*^BMI– 2.982^*^CP ratio + 0.027^*^AST)]}. The nomogram of the CP ratio model for discriminating NASH is shown in Figure 3.

**Table 3:** Univariate and multivariable analysis.

**Parameters**	***p***	**Crude OR**	**95% CI**		***p***	**Adjusted OR**	**95% CI**	
			**Lower**	**Upper**			**Lower**	**Upper**
Male sex	0.020	3.437	1.220	9.689				
CP ratio	0.022	0.134	0.024	0.746	0.006	0.051	0.006	0.432
BMI	0.011	1.206	1.044	1.393	0.007	1.245	1.062	1.460
ALT	0.043	1.012	1.000	1.023				
AST	0.043	1.027	1.001	1.054	0.034	1.028	1.002	1.054
UA	0.026	1.006	1.001	1.012				
TG	0.043	2.362	1.028	5.426				

*ALT, alanine transferase; AST, aspartate transferase; CI, confidence interval; CP, ceruloplasmin; TG, triglyceride; OR, odds ratio; UA, uric acid*.

### The Performance of the CP Ratio Model in Discriminating NASH From NAFLD Patients

**Figure 4:**
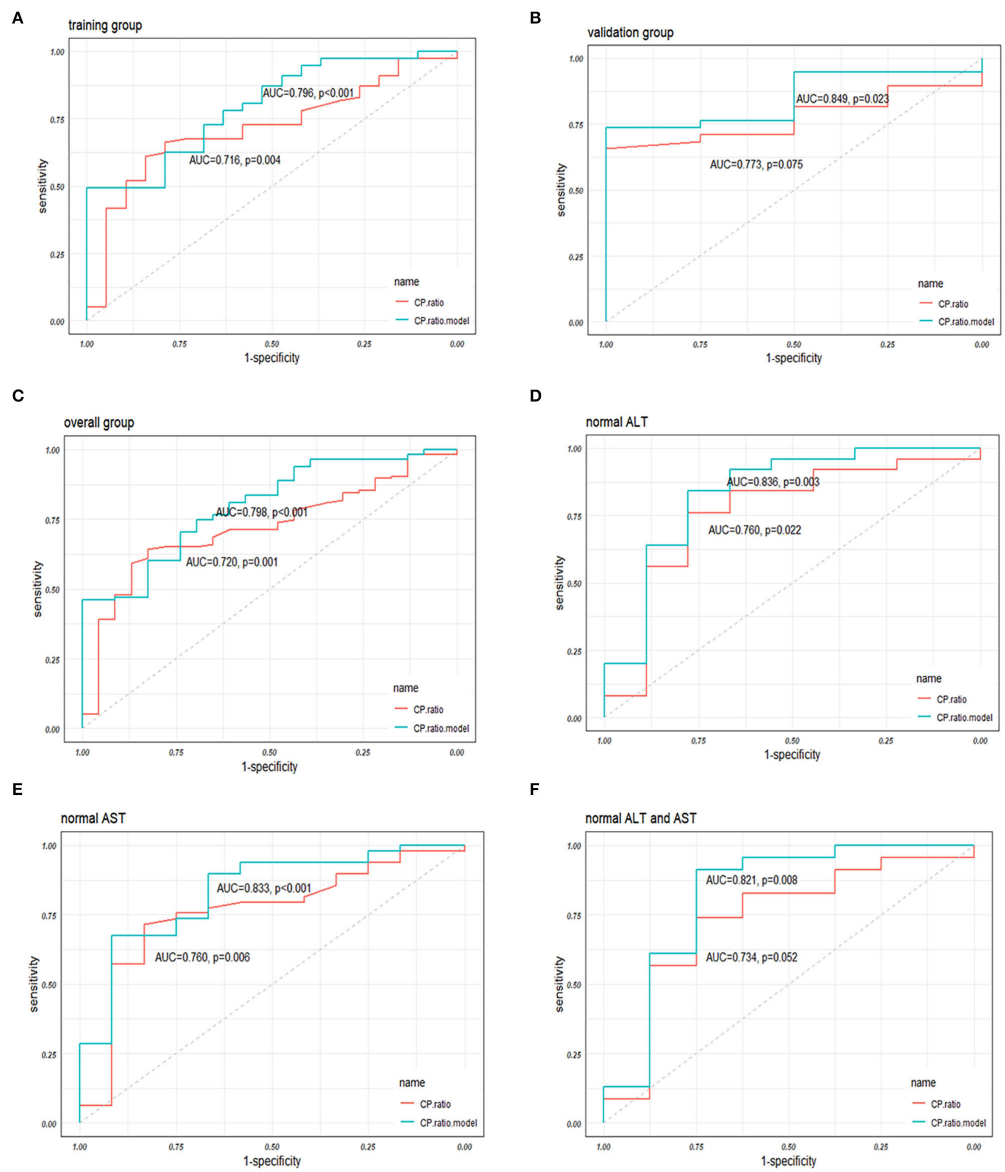
The performances of the CP ratio and the CP ratio model in discriminating NASH in **(A)** the training, **(B)** validation, and **(C)** overall groups, and in patients with **(D)** normal ALT, **(E)** AST, and **(F)** both normal levels. Abbreviations: ALT, alanine transferase; AST, aspartate transferase; AUC, area under the receiver operator characteristic curve; CP, ceruloplasmin; NASH, nonalcoholic steatohepatitis; ROC, receiver operator characteristic curve.

A receiver operator characteristic curve (ROC) analysis was performed to evaluate the performance of the CP ratio and model in discriminating NASH from NAFLD patients. In the training group, the area under the ROC (AUC) of CP ratio alone was 0.716 (0.597–0.835) with a cutoff of 1.20, while the sensitivity and specificity were 84.2 and 61.0%, respectively. The AUC of the CP ratio model was 0.796 (0.694–0.899) with a cutoff of 0.78, while the sensitivity and specificity were 72.7 and 68.4%, respectively (Figure 4A). In the validation group, the CP ratio model was validated with an AUC of 0.849 (0.713–0.984, Figure 4B). In the overall group, the AUC of the CP ratio model was 0.798 (0.708–0.888, Figure 4C). The performance of the CP ratio model was better than the CP although not significant (*p* > 0.05).

The overall patients were stratified according to the upper limit of normal serum ALT and/or AST levels, while 34 (24.6%), 61 (44.2%), and 31 (22.5%) patients were in the normal range of ALT, AST, and both, respectively. The AUCs of CP ratio alone for NASH prediction in NAFLD patients with normal ALT or AST, or both were 0.760 (0.560–0.960), 0.760 (0.605–0.915), 0.734 (0.516–0.951), respectively, while the AUCs of CP ratio model were 0.836 (0.659–1.000), 0.833 (0.705–0.962), and 0.821(0.612–1.000), respectively (Figures 4D–F). The AUCs of the CP ratio model were greater than those of the CP value in patients with normal ALT, AST, or both (*p* > 0.05), however, statistical significance was not observed.

**Figure 5:**
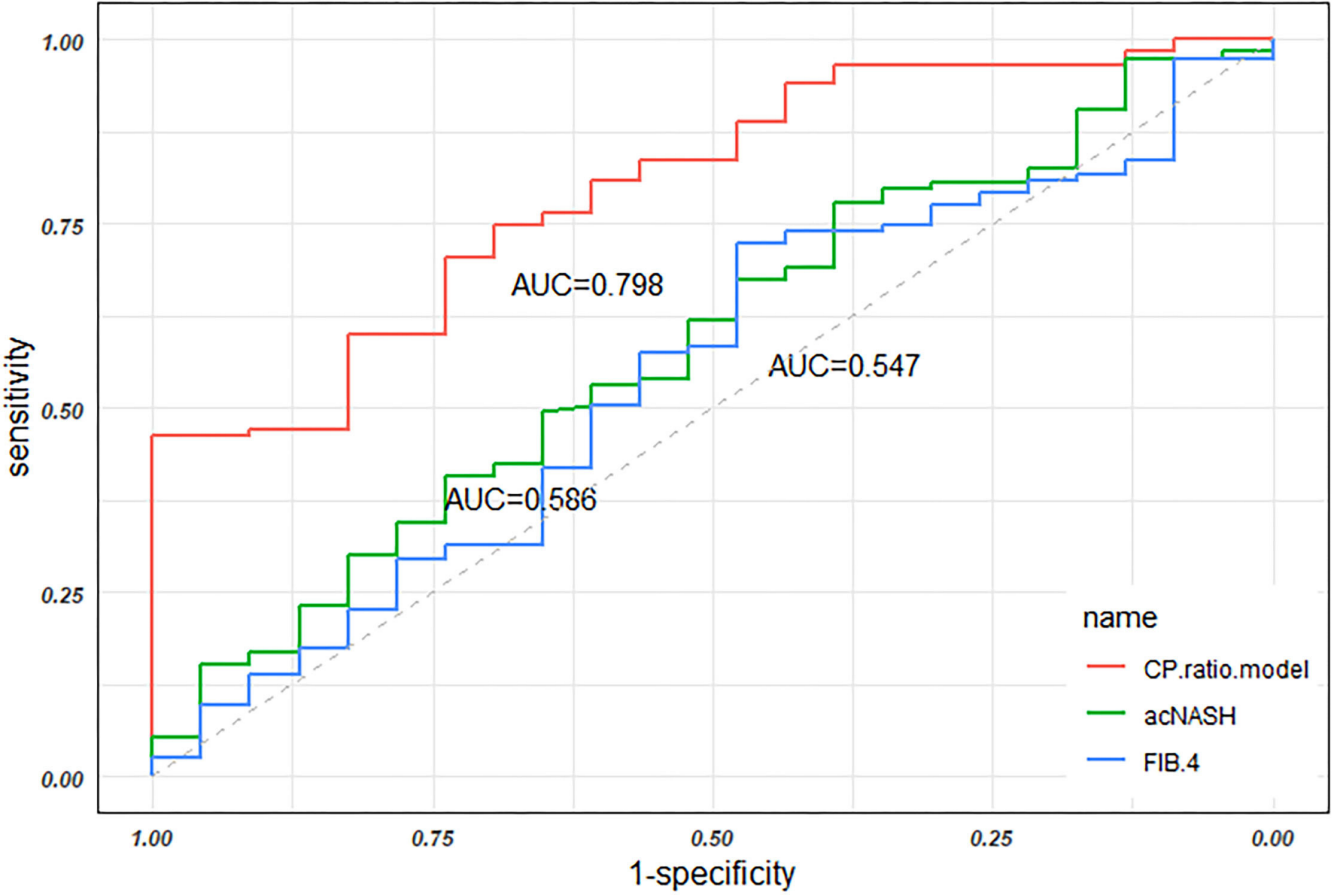
The AUCs of the CP ratio model, the acNASH index, and FIB-4 in discriminating NASH in the overall groups. Abbreviations: AUC, area under the receiver operator characteristic curve; CP, ceruloplasmin; NASH, nonalcoholic steatohepatitis; ROC, receiver operator characteristic curve.

The FIB-4 constituted by age, ALT, AST, and PLT (29) was calculated in the present study. The AUC of FIB-4 was 0.547 (0.417–0.677), which is significantly lower than the CP ratio model by the Delong test (*p* = 0.004, Figure 5). Recently, a novel model named acNASH index was proposed to identify NASH patients, which was computed through the serum AST divided by the creatine and then multiplied by 10 (30). The AUC of the acNASH index in the present study was significantly lower than that of the CP ratio model [0.586 (0.459–0.712) *vs*. 0.798 (0.708–0.888), *p* = 0.002, Figure 5].

## Discussion

The present study enrolled biopsy-proven patients with NAFLD without T2DM and showed that the CP ratio was inversely correlated to the histological severity of NAFLD, the presence of NASH, and hepatic iron overload. Despite the CP ratio alone having a certain value in discriminating NASH from patients with NAFLD, the developed CP ratio model showed better performance, even in those with normal aminotransferase levels.

The priority of the present study is the development and validation of the CP ratio model with a good performance for discriminating NASH. A few models have been established to identify NASH so far, while these models have not been extensively validated yet. The Gholam's model, comprised of serum AST and the presence of T2DM, was proposed to have an AUC of 0.82 with sensitivity and specificity of 76 and 66% for predicting NASH (9), however, the studied population of severe obesity with a mean BMI of 55 ± 12 Kg/m^2^ and predominate female with a proportion of 83% limit its application in the general population. The NashTest score, including 13 parameters, had an AUC of 0.79 with sensitivity and specificity of 33 and 97% (10). However, it is not suitable for clinic application as it is highly complex in the calculation. These disadvantages are also presented in the Nice model (11) and the NI-NASH-DS model (12).

Recently, Wu et al. developed an acNASH index that combined serum creatine and AST levels for identifying NASH with AUCs of 0.805–0.818 (30). However, its performance was unsatisfactory in the present study with a significantly lower AUC than the CP ratio model (0.586 *vs*. 0.798, *p* = 0.002). Concerning the similar ethnicity of their derivation group with our overall group, the possible reason for the difference is the high proportion (28.5%) of T2DM patients in their derivation group. While in the present study the patients with T2DM were excluded due to the mutually detrimental relationship between NAFLD and T2DM. The histopathology of NAFLD patients with T2DM is more severe and recognizable compared to those without T2DM. The present study aimed to examine the predictive value of CP in the diagnosis of NASH at a relatively earlier stage of NAFLD. Thus, NAFLD patients with T2DM were ruled out.

In the present study, there were 24.6, 44.2, and 22.5% of patients with NAFLD were in the normal range of ALT, AST, and both levels, respectively. Multiple existing studies have manifested that the prevalence of histological NASH with normal ALT in NAFLD patients varies from 10.7 to 59.0%, and the risk of fibrosis stage ≥2 of patients with normal ALT is similar to those with elevated ALT (31–33). Because the golden standard liver biopsy is not readily accepted by patients with NAFLD with normal transferases, the non-invasive approach is an indispensable alternative. The LACSNA model was reported with an AUC of 0.70 for discriminating NASH with both normal ALT and AST levels, but it is highly complex (34). The G-NASH model with an AUC of 82.9% for identifying NASH patients with normal ALT, whereas the serum Golgi protein 73 incorporated in this model is unfeasible in the clinic (35). While a simpler, more convenient model named the CP ratio model was derived and validated in the present study. More importantly, the CP ratio model has good performance in discriminating NASH not only from NAFLD patients with normal ALT or AST levels but also from both normal ALT and AST levels.

In line with existing literature (36), the present study found a trend that the CP ratio decreased along with the increasing severity of steatosis. This can be explained by the lowered hepatic copper concentration of NAFLD (37). Copper deficiency results in dysregulated lipid metabolism through promoting lipogenesis (38). Further, reduced liver copper induces glucose intolerance and insulin resistance (39). These factors drive the accumulation of hepatic lipids. A previous study reported that in NAFLD patients the hepatic copper concentration was inversely associated with the severity of steatosis (40). *In vivo*, an 8-week copper-deficiency diet can induce marked hepatic steatosis in rats, while a normal or copper-enrichment diet did not (37). Meanwhile, CP deficiency results in intracellular iron accumulation due to its critical role in the efflux of cellular ferrous iron (41) and the stability of ferroportin (42). Subsequently, the spontaneous oxidation of ferrous iron accumulated in hepatocytes triggers oxidative stress by Fenton reaction, leading to immune cell activation and hepatocellular injury (43) and initiates ferroptosis (44). Accordingly, NAFL progresses to NASH. In addition, hepatic iron overload is known pathogenesis of fibrosis (45). Recently, a study found that patients with NAFLD who carried the mutation of CP presented with more severe liver siderosis and fibrosis (46). The transcriptomics analysis of animals found that a low copper diet can upregulate the expression of inflammation and hepatic stellate cell activation associated genes (47). In brief, it is reasonable that the CP ratio was inversely associated with the histological severity of NAFLD.

Another interesting discovery is that the CP ratio was significantly lower in patients with NASH than that in patients with NAFL in the present study, whereas the CP has been deemed as an acute-phase protein which will increase under the condition of inflammation (48). One reason might be the endoplasmic reticulum (ER) stress induced by excessive lipotoxic lipid and its products (49). The ER stress can affect the process of synthesis, folding, and trafficking of CP protein in hepatocytes (50). The other reason could be the copper availability which is significantly lower in patients with NASH than in those with NAFL (37). Copper deficiency leads to the secretion of apo-CP which is ferroxidase inactivity (51) and rapidly catabolized in about 5 h, whereas the holo-CP is in 5.5 days (15). Thus, although NASH upregulates the expression of CP, the secreted apo-CP would be degraded soon, leading to an inverse association of CP and NASH.

We acknowledge that in the present study there are also several limitations. First, the CP ratio model is derived from patients with NAFLD without T2DM. The performance of this model in the general NAFLD population is unsure and needs to test further. Second, the data of patients come from two different hospitals, however, this well-developed model is only internally validated. It still needs to be validated externally and extensively.

Taken together, our data suggested that the standardized CP ratio was inversely associated with the severity of NAFLD, the presence of NASH, and hepatic iron deposition. The novel CP ratio model may serve as a simple tool for identifying patients with NASH from those with NAFLD, even in those with normal aminotransferases. The application of the novel model might reduce the need for liver biopsy in the clinic.

## Data Availability Statement

The raw data supporting the conclusions of this article will be made available by the authors, without undue reservation.

## Ethics Statement

The studies involving human participants were reviewed and approved by the Institutional Ethics Committee of Shanghai Ruijin Hospital and Zhongshan Hospital. The patients/participants provided their written informed consent to participate in this study.

## Author Contributions

Study concept and design: LC, TL, and XZ. Acquisition of data: QW, DZ, MZ, MW, and ML. Analysis and interpretation of data: LC, QW, and PC. Drafting of the manuscript: QW and DZ. Critical revision of the manuscript: LC, TL, XS, XZ, and MW. All authors contributed to the article and approved the submitted version.

## Funding

This work was supported in part by the National Natural Science Foundation of China (81800510), Ruijin Hospital Research Initiative Project (2019ZX01), and Shanghai Science and Technology Commission, Science and Technology Innovation Action Plan Sailing Project (21YF1440400 and 18YF1415900).

## Conflict of Interest

The authors declare that the research was conducted in the absence of any commercial or financial relationships that could be construed as a potential conflict of interest.

## Publisher's Note

All claims expressed in this article are solely those of the authors and do not necessarily represent those of their affiliated organizations, or those of the publisher, the editors and the reviewers. Any product that may be evaluated in this article, or claim that may be made by its manufacturer, is not guaranteed or endorsed by the publisher.
