# Supplementary material for: A Novel Non-Invasive Approach Based on Serum Ceruloplasmin for Identifying Non-Alcoholic Steatohepatitis Patients in the Non-Diabetic Population
Source: Front Med (Lausanne). 2022 Jun 20;9:900794. doi: 10.3389/fmed.2022.900794 (PMC9252518; doi:10.3389/fmed.2022.900794)
Supplement: Supplementary file 1 [file Data_Sheet_1.docx]

Supplementary Material

# Supplementary Tables

**Supplementary Table 1. The clinical and demographic characteristics of patients in the training and validation groups**

| **Variables** | **Overall (n=138)** | **Training group (n=96, 70%)** | **Validation group (n=42, 30%)** | **P value** |
| --- | --- | --- | --- | --- |
| Male, n (%) | 84 (60.9) | 63 (65.6) | 21 (50.0) | .084 |
| age, (years) | 39.00 (33.00-54.25) | 39.00 (33.00-50.75) | 47.50 (34.75-58.50) | .065 |
| BMI, (Kg/m^2^) | 27.40 (24.67-30.88) | 27.68 (25.11-31.44) | 26.79 (23.93-29.67) | .182 |
| Hypertension, n (%) | 46 (33.3) | 30 (31.2) | 16 (38.1) | .433 |
| MetS, n (%) | 65 (47.1) | 46 (47.9) | 19 (45.2) | .722 |
| CP, (mg/L) | 211.00 (186.00-236.63) | 207.65 (184.55-232.50) | 214.00 (189.85-240.40) | .269 |
| decrease CP, n (%) | 28 (20.3) | 21 (21.9) | 7 (16.7) | .484 |
| CP ratio | 1.19 (1.03-1.43) | 1.19 (1.03-1.43) | 1.20 (1.02-1.45) | .655 |
| ALT, (IU/L) | 88.00 (50.75-128.25) | 93.00 (51.00-127.75) | 70.50 (45.50-132.00) | .551 |
| AST, (IU/L) | 48.00 (32.00-66.00) | 48.00 (32.00-64.75) | 47.00 (31.75-69.00) | .930 |
| AKP, (IU/L) | 74.00 (62.75-89.25) | 76.00 (63.00-92.00) | 70.50 (62.00-89.00) | .532 |
| GGT, (IU/L) | 60.00 (35.00-87.00) | 62.50 (36.25-86.00) | 56.00 (34.00-91.75) | .411 |
| UA, (μmol/L) | 412.00 (324.75-487.08) | 426.60 (337.50-490.08) | 384.90 (302.45-474.50) | .052 |
| Creatine, (μmol/L) | 76.50 (65.00-89.50) | 77.00 (65.00-88.50) | 71.00 (63.00-94.00) | .673 |
| TG, (mmol/L) | 1.70 (1.22-2.20) | 1.72 (1.26-2.29) | 1.53 (1.12-2.16) | .254 |
| CHOL, (mmol/L) | 4.92 (4.19-5.59) | 4.92 (4.18-5.61) | 4.90 (4.22-5.57) | .421 |
| HDL, (mmol/L) | 1.06 (0.96-1.22) | 1.05 (0.94-1.21) | 1.10 (0.98-1.27) | .299 |
| LDL, (mmol/L) | 3.00 (2.60-3.68) | 3.00 (2.58-3.68) | 2.99 (2.64-3.69) | .727 |

Abbreviations: AKP, alkaline phosphatase; ALT, alanine transferase; AST, aspartate transferase; BMI, body mass index; CHOL, cholesterol; CP, ceruloplasmin; GGT, gamma-glutamyl transferase; HDL, high density lipoprotein; LDL, low density lipoprotein; MetS, metabolic syndrome; TG, triglyceride; UA, uric acid.

**Supplementary Table 2. The histopathological features of patients in the training and validation groups**

| **Variables** | **Overall (n=138)** | **Training group (n=96, 70%)** | **Validation group (n=42, 30%)** | **P value** |
| --- | --- | --- | --- | --- |
| Steatosis, n (%) |  |  |  | - |
| 0 | 4 (2.9%) | 4 (4.2%) | 0 (0.0%) |  |
| 1 | 51 (37.0%) | 32 (33.3%) | 19 (45.2%) |  |
| 2 | 51 (37.0%) | 38 (39.6%) | 13 (31.0%) |  |
| 3 | 32 (23.2%) | 22 (22.9%) | 10 (23.8%) |  |
| Steatosis 1-3, n (%) | 134 (97.1%) | 92 (95.8%) | 42 (100%) | .429 |
| Steatosis 2-3, n (%) | 83 (60.1%) | 60 (62.5%) | 23 (54.8%) | .393 |
| Steatosis 3, n (%) | 32 (23.2%) | 22 (22.9%) | 10 (23.8%) | .909 |
| Activity, n (%) |  |  |  | - |
| 0 | 2 (1.4%) | 2 (2.1%) | 0 (0.0%) |  |
| 1 | 16 (11.6%) | 13 (13.5%) | 3 (7.1%) |  |
| 2 | 45 (32.6%) | 33 (34.4%) | 12 (28.6%) |  |
| 3 | 36 (26.1%) | 26 (27.1%) | 10 (23.8%) |  |
| 4 | 39 (28.3%) | 22 (22.9%) | 17 (40.5%) |  |
| Activity 1-4, n (%) | 136 (98.6%) | 94 (97.9%) | 42 (100%) | 1.000 |
| Activity 2-4, n (%) | 120 (87%) | 81 (84.4%) | 39 (92.9%) | .173 |
| Activity 3-4, n (%) | 75 (54.3%) | 48 (50%) | 27 (64.3%) | .121 |
| Activity 4, n (%) | 39 (28.3%) | 22 (22.9%) | 17 (40.5%) | .035 |
| NASH, n (%) | 115 (83.3%) | 77 (80.2%) | 38 (90.5%) | .136 |
| Fibrosis, n (%) |  |  |  | - |
| 0 | 33 (23.9%) | 24 (25.0%) | 9 (21.4%) |  |
| 1 | 32 (23.2%) | 21 (21.9%) | 11 (26.2%) |  |
| 2 | 52 (37.7%) | 34 (35.4%) | 18 (42.9%) |  |
| 3 | 16 (11.6%) | 13 (13.5%) | 3 (7.1%) |  |
| 4 | 5 (3.6%) | 4 (4.2%) | 1 (2.4%) |  |
| Fibrosis 1-4, n (%) | 105 (76.1%) | 72 (75%) | 33 (78.6%) | .651 |
| Fibrosis 2-4, n (%) | 73 (52.9%) | 51 (53.1%) | 22 (52.4%) | .936 |
| Fibrosis 3-4, n (%) | 21 (15.2%) | 17 (17.7%) | 4 (9.5%) | .218 |
| Fibrosis 4, n (%) | 5 (3.6%) | 4 (4.2%) | 1 (2.4%) | .983 |
| Positive Perl's staining, n (%) | 42 (30.4) | 32 (33.3) | 10 (23.8) | .263 |

Abbreviations: NASH, nonalcoholic steatohepatitis.
